# Moving together—benefits of a 12-week online dance training intervention on static and dynamic postural stability and gait speed in older women: an exploratory pre-post study

**DOI:** 10.3389/fspor.2024.1384387

**Published:** 2024-07-04

**Authors:** Rasmus Kopp Hansen, Elizabeth Jochum, Ditte Egholm, Morten Villumsen, Rogerio Pessoto Hirata

**Affiliations:** ^1^ ExerciseTech, Department of Health Science and Technology, Aalborg University, Aalborg, Denmark; ^2^ Respiratory and Critical Care Group, Department of Health Science and Technology, Aalborg University, Aalborg, Denmark; ^3^ Department of Communication and Psychology, Aalborg University, Aalborg, Denmark; ^4^ Center for Orthopedic Rehabilitation and Head of Center for Fall Prevention, Marselisborg Rehabilitation Center, Aarhus, Denmark; ^5^ Department of Health Science and Technology, Faculty of Medicine—Pain and Motor System Plasticity, Aalborg University, Aalborg, Denmark

**Keywords:** online dance training, physical activity, postural stability, gait speed, older adults

## Abstract

**Background:**

Physical inactivity negatively affects gait performance and postural stability in older adults resulting in a higher risk of fall accidents. Previous research has shown that in-person dance training improves various aspects of balance and lower-extremity function, however, little is known about the potential benefits of dance training delivered online on variables used for fall risk stratification in older adults. We aimed to explore the benefits of a 12-week online dance training intervention on static and dynamic postural stability and gait speed in older women.

**Methods:**

Forty-five older adults (44 women) were included in this exploratory pre-post study. The 12-week dance intervention consisted of two weekly 60-min classes in contemporary (improvisation) and salsa dance delivered online through Zoom video calls. Static and dynamic postural stability was assessed using the center of pressure (CoP) area and velocity (force platform), and the Mini Balance Evaluation Systems Test (Mini-BESTest), respectively. 10-m gait speed was measured using photo gates. Before and after comparisons were performed using paired sample *t*-tests.

**Results:**

Thirty-two older women completed the study. There were no significant changes for static postural stability assessed by CoP area or velocity (*P* ≥ 0.218, Cohen *d* ≤ 0.234). The Mini-BESTest total score was significantly improved at post-intervention (23.88 ± 3.01) compared to baseline (22.56 ± 1.41, *P* = 0.007, *d* = 0.52). 10-m gait speed was significantly faster at post-intervention (1.68 ± 0.25 m/s) compared to baseline (1.57 ± 0.22 m/s, *P* < 0.001, *d* = 0.737).

**Discussion:**

Although some caution is needed due to the uncontrolled study design, the results indicate that online dance training has a small effect on static postural stability but may be beneficial for gait speed and in particular dynamic postural control among older women. While the absolute increase in gait speed suggests limited clinical relevance, the change in Mini-BESTest score may reflect a clinically meaningful enhancement of dynamic postural control.

## Introduction

Physical inactivity negatively affects gait performance and postural stability in older adults, which results in a higher risk of fall accidents. Medical costs related to falls in older adults were estimated to be approximately $50 billion per year in the USA alone (1). This is a concerning public health problem, as around 30% of the older adult population is expected to experience an accidental fall within a one-year time period (2). Common outcomes from fall accidents in older adults are: head injuries (3), fear of falling (4), loss of physical independence (5), increased healthcare utilization (6), and mortality (7), among others.

Falls are multifactorial in nature, however, some of the major modifiable risk factors identified include impaired mobility and gait, balance, and muscle strength deficits (8). In the current world guidelines for fall prevention, dynamic gait evaluations are used for stratification of older adults in relation to fall risk (9). Low gait speed has previously been correlated with increased risk for accidental falls in community-dwelling older adults (10), a risk that is specifically amplified when gait speed is lower than 1 m/s (11). Moreover, the capacity to carry out concurrent postural tasks is paramount for maintaining balance in daily life activities, since its deficit may increase the risk of accidental falls in this population (12). Such capacity may also involve the prioritization of one of the tasks over the other, which in postural control studies is often referred to as the “postural first” principle, when the focus on maintaining the static posture stability overcomes the performance of the supra-postural task (13).

Evidence from recent systematic reviews strongly suggests that physical activity and exercise programs are among the most effective strategies for reducing the rate of falls among older adults (14, 15), an effect that may relate to the improvement of gait and balance stability deficits (16). Dancing is an enjoyable physical activity that can be tailored to different cultural and aging backgrounds, and the physical needs of older adults, and has the potential to enhance emotional, psychological, and social well-being (17). According to systematic reviews, dance is a valuable health promoting intervention for supporting healthy aging, for example by improving physical function performance (18), and mitigating fall-related risk factors (19). Studies using in-person dance training have reported improved medial-lateral balance (20), reduced dependency of visual input for balance maintenance (21), reduced attention demands during postural tasks (20), and improved lower extremity function in older adults after as little as one weekly dance class for 12 weeks (22). Concerning falls, the current Cochrane Review did not find enough evidence to determine the effects of dance programs on the rate of falls in community-dwelling older adults, although Tai Chi, which possesses several similarities with dance, seems to reduce the risk of falls (15). In contrast, another systematic review and meta-analysis recently reported that dance-based mind-motor activities were significantly associated with reduced risk of falling and rate of falls among healthy older adults (23).

In terms of specific dance styles, Meron et al. (24) reported that 12 months of twice-weekly 1-h sessions of social dancing (folk or ballroom dance) did not prevent falls or associated physical and cognitive fall-related risk factors. In contrast, Granacher et al. (25) observed enhanced dynamic postural control and a tendency for improved static postural control following 8 weeks of twice-weekly in-person salsa dance in healthy older adults. Moreover, a randomized controlled trial from our group recently indicated that a combination of in-person and web-based salsa dance significantly reduces the number of fall accidents in older adults (26). The benefits of salsa dance for dynamic postural control may not be surprising as salsa incorporates frequent changes in direction and with the dance steps performed on the toes of the feet which may effectively challenge the maintenance of balance (25). Contemporary dance is another dance style that may appeal to the older adult population as it can be adapted to be a low-impact physical activity and does not require any specific baseline skill level or physical fitness level (27). Contemporary dance typically consists of elements of improvisation to feelings or the music, individually or together with others, and includes elements of aerobic exercise, movements targeted enhancement of flexibility, and body weight exercises to strengthen both large and small muscle groups (28). Britten et al. (29) reported that 8 weeks (2 sessions a week) of in-person contemporary dance had positive effects on the modification of physical and psychological risk factors for falling in community-dwelling older adults, including an improvement in dynamic balance and mobility.

Taken together, although a growing pool of studies supports the benefits of in-person dance training (e.g., salsa and contemporary dance) on healthy aging, including the risk of falls, a limited number of studies conducted in older adults have explored the potential benefits of dance training delivered digitally (online) (30–32). Such online-based dance training may be particularly relevant in times of restricted access to physical activity, such as during the recent COVID-19 pandemic. Moreover, while home-based and digital training have previously proven to be effective for combating physical inactivity among this population (33), less is known about whether this type of training may improve variables used for fall risk stratification in older adults, such as gait speed and postural stability. We recently reported that a 12-week online dance training intervention, the *Moving Together* (MT) program, consisting of salsa and contemporary (improvisation) dance, was associated with improvements in mental health and various aspects of functional fitness in older women (34). Therefore, the aim of this study was to explore the benefits of the MT program on static and dynamic postural stability and gait speed. Static postural stability is here defined as the ability to hold an upright position as still as possible over a stable base of support, while dynamic postural stability is the ability to maintain balance, i.e., keeping the body's center of mass vertical projection within the base of support, when changes in the base of support occur (35). Throughout the evaluation of the intervention's impact on these specific physical aspects, the study also seeks to contribute valuable insights into the literature on the potential benefits of online dance training in the functional performance of older adults. The hypothesis was that static postural stability, dynamic postural stability, and gait speed would improve after 12 weeks of the online MT intervention.

## Materials and methods

### Participants

Forty-five older adults (44 women) were included in this uncontrolled exploratory pre-post study (Table 1). The sample size was based on an *a priori* power analysis using the G*Power software (ver. 3.1, Heinrich-Heine-Universität Düsseldorf, Düs-seldorf, Germany) for a single-group pre-post study design, with an accepted type I error rate of 0.05, a type II error rate of 0.10 (90% statistical power), and with an estimated dropout of 20%. Considering an expected effect size (Cohen's *d*) of 0.55, it was estimated that 37 participants would be required to detect a statistically significant change in the primary outcome measure of the MT intervention (i.e., self-reported loneliness), as detailed elsewhere (34). Notably, the sample size calculation was based on another outcome than presented in this study, and thus the current study may be underpowered to detect significant changes in postural control and gait speed. The participants were recruited through advertisements posted at activity centers for older adults in Aalborg Municipality, through information meetings, by advertisement in the press, and through flyers handled out in public spaces. To ensure inclusivity, only two inclusion criteria were used: (I) being 65 years or older, and (II) being able to speak and understand Danish. Individuals who were unable to stand and walk independently were excluded from participation. No criteria were used in terms of prior dance experience. The study was approved by the North Denmark Region Committee on Health Research Ethics (N-20220045) and conducted in accordance with the Declaration of Helsinki.

**Table 1:** Baseline participant demographics (*N* = 45).

Sex, W/M	44/1
Age, year	74.0 ± 5.3
Height, m	1.62 ± 0.58
Body mass, kg	69.7 ± 13.0
BMI, kg/m^2^	26.4 ± 4.9

W, women; M, men; BMI, body mass index. Values are *n*, or mean ± SD.

### Dance training intervention

The study builds upon a previous randomized controlled trial from our group (36) demonstrating that regular fitness circuit training and a combination of in-person and online salsa dance significantly reduced the number of fall accidents in older adults (26). In this separate study, the new and unique aspect was the completely online/digital implementation of dance training in practice (in the municipality). Specifically, the MT program consisted of two weekly dance classes lasting 60 min each, consisting of contemporary (improvisation dance) (Day 1) and salsa (Day 2) performed for 12 weeks. Both types of classes followed the same general structure consisting of a warm-up, main session, and cool-down. For the contemporary classes, however, the warm-up and cool-down specifically focused on breathing exercises. Common for both types of classes was that a series of movements were introduced and practiced to music. (I) The movement language of Laban's choreological practice (37) was used to inspire and create variations of movement during the contemporary dance classes, including verbalizing movement such as different spatial levels, rhythms, moving with your own kinesphere or in the general space. A guided dance improvisation approach was used, with attention towards inclusive dance practice where participants could adapt the dance movements to their physical needs. For example, if some participants needed to take a break more often than others, they were encouraged to continue dancing with their upper body. One specific movement example from the classes was to do a small leap and pretend to land on a waterlily. The landing had to be soft and soundless, and the participants were given the imagery to not destroy the waterlily. When landing on one leg, participants were instructed to hold the position for as long as possible, while at the same time encouraged to maintain creative dance movements with their upper body, thus challenging their dynamic balance. A relative “%-system” approach was used by which participants were encouraged to “listen to their own needs”, and how they felt regarding their own movement abilities and “energy level” for that specific day without necessarily having to copy what other participants did, such as dancing in the same pace or reaching as high as others. Each class started sitting on chairs with slow mobilizing movements of the torso. Here, it was introduced from the beginning that sitting still on a chair performing slow movements was between 0% and 10% of participants' maximum output (symbolized as 100%). The dance teacher used this principle from the start to the end of the classes. Every class was structured by starting slow (0%–10%) and then continuously building up the intensity as the class progressed by using larger and faster movements and by using more space to travel across. The increase in intensity was supported by faster-paced music opposite to the slower-paced music used at the beginning of the class. The music playlist was the same for 3–4 classes before it was changed. (II) The salsa classes were built upon techniques from Cuban salsa; participants had a progressive introduction to the technique and the expressive aesthetics of the dance style. Over time, the class included dancing with faster pace. The movements involved basic steps forward and backward and turns for both sides while keeping the movement paced to the music. All salsa classes were tailored so that they could be performed individually keeping up with the online format.

**Figure 1:**
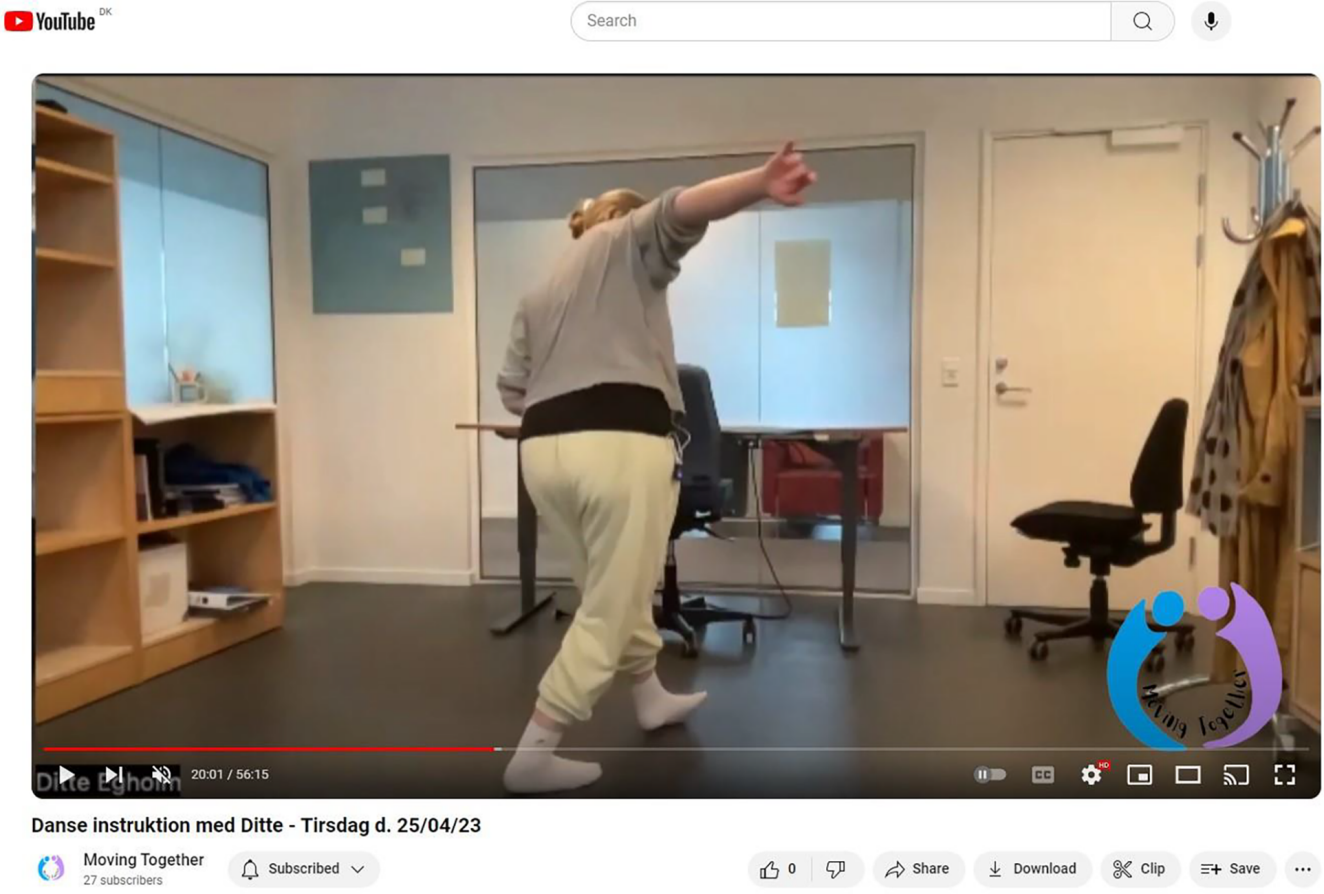
Image from an online dance video (contemporary dance) posted on the designated YouTube channel.

Both the contemporary and salsa classes were delivered by experienced professional dance instructors online through video calls using the Zoom meeting application (Zoom Video Communications, Inc., San Jose, CA, USA). For flexibility purposes, subjects could choose to attend the online classes either from home, or at one of four activity centers in the municipality in a group setting. Each online class was recorded and uploaded to a designated YouTube channel connected to the project after each class.^1^ The YouTube channel resource (Figure 1) was made available for all participants and the public, where participants could review and replay the classes if they were unable to attend on the day. Participants were instructed to dance at least once per week but were encouraged to attend twice weekly. To promote engagement and social connection between the participants, a 60-min in-person salsa dance class was conducted once every month with physical presence by both the instructor and the participants (up to three sessions in total per subject). Given the exploratory study design and focus on implementation in practice, no restrictions were made on the participants regarding participation in other dance or training activities during the intervention period.

### Measurements

All measurements were performed immediately prior to (baseline) and following 12 weeks of dance training and were carried out at local activity centers in Aalborg Municipality. The same experienced investigator was present at all tests and either performed the testing or provided supervised assistance to two student helpers.

#### Static postural stability

Static postural stability during a quiet standing task was assessed for 30 s both with and without a dual-task. A high-resolution three-dimensional, four-channel, force platform (PLUX Wireless Biosignals S.A., Lisboa, Portugal) sampled at 1,000 Hz was placed 1.5 m from a wall. A circle (*d* = 5 cm) was placed on the wall in front of the participants at approximately eye height. At baseline, participants were instructed to stand on the force platform in a self-determined, comfortable position with the distance between the feet measured with a non-elastic measuring tape. The distance between the feet was then replicated at the post-test. Participants were instructed not to move during the 30-s recording period. Measurements were calibrated prior to each participant by recording a 10-s period with zero load on the force platform. The center of pressure (CoP) data from the force platform was extracted from the horizontal forces and filtered digitally with a low-pass filter and a 20 Hz cut-off frequency. The static postural stability was then quantified from the CoP data via the 95% prediction ellipse CoP area and resultant mean CoP velocity (38). 3×single- and 3×dual-task recordings were performed. During the dual-task conditions, a mathematic task was used as a supra-postural task, by which participants were instructed to count backwards of “minus 7”, starting from a random number between 0 and 500 (e.g., 40, 33, 26 etc.). The starting number for the 3 dual-tasks remained constant between visits. The mean of the 3 single-tasks and the 3 dual-tasks for both variables were used for analyses.

#### 10-m gait speed

Gait speed was determined using photo gates (Brower Timing Systems, Draper, UT, USA) placed in a corridor 10 m apart. Participants were instructed to walk as fast as possible without running (39) (“Please walk as quickly as you can without running to the end of the corridor”). Participants started walking two meters before the start line so that gait speed did not include the acceleration time, with an additional 2-m walk-out after the participants crossed the finish line. A total of 6 walks were performed (6 × 10 m), with a self-determined break in between walks. For the last 3 10-m walks, participants were instructed to perform the same mathematical dual-task as for the assessment of postural control, yet with other starting numbers. For these dual-tasks, participants were instructed to walk as fast as possible without running, while counting as correctly as possible. The mean of the 3 single-tasks and the 3 dual-tasks, respectively, were used for analyses. For comparison with previous studies, the time to complete the 10-m walk was converted to speed (m/s).

#### Dynamic postural stability (Mini-BESTest)

Dynamic postural stability was determined using the Mini Balance Evaluation Systems Test (Mini-BESTest) (40). The Mini-BESTest has been extensively used in clinical settings to evaluate anticipatory and reactive postural responses and gait dynamics (40) due to its high construct validity and ability to discriminate between groups with respect to fall risk in older adults (41). The Mini-BESTest consists of 14 items related to domains of anticipatory transitions; reactive postural control; sensory orientation; and dynamic balance during gait. The procedures for administration and scoring of the Mini-BESTest were standardized as described in (42). For each item, a score from 0 (severe) to 2 (normal) was provided. Individual item scores were then summated and reported as the Mini-BESTest total score (out of the maximum attainable 28 points) (42).

### Statistics

Statistical analyses were performed using SPSS software (version 27; IBM, Armonk, New York). Normal distribution of data was confirmed with the Shapiro-Wilk Test, with data reported as mean ± SD. Statistical significance was set *a priori* at *P* < 0.05. Before and after comparisons on outcomes were performed using paired sample *t*-tests (two-tailed). We also calculated standardized effect sizes (Cohen *d*) to determine the magnitude of change from baseline to post-intervention, with the following thresholds used for interpretation: small (>0.2), moderate (>0.5), and large (>0.8).

## Results

Of the 45 participants included at baseline, 13 dropped out, so that *n* = 32 in total (all women) were included for analyses. All 32 participants were included in analyses irrespective of where and how they attended. Reasons for dropout included non-study related health issues (*n* = 5), lack of motivation (*n* = 4), COVID-19 infection (*n* = 1), finding it too physically demanding to complete the dance sessions (*n* = 2), and finding it uncomfortable being the only man (*n* = 1). There were no study-related incidents or falls during the intervention.

### Static postural stability

Results for static postural stability are shown in Table 2. Due to corrupted/erroneous data for three participants, analyses are based on *n* = 29. There were no significant changes from baseline to post-intervention for either single- or dual-task CoP area or velocity (*P* ≥ 0.218). The mathematical performance during the dual-task is presented in Table 3. There were no significant changes in the mathematical performance during the dual-task, either when expressed as the total number of counts, or as the relative or absolute number of correct counting's (*P* ≥ 0.28).

**Table 2:** Static postural stability assessed via area and velocity of center of pressure (CoP) during 30 s of quiet standing with and without a dual mathematical load at baseline and after 12 weeks of online dance training (*n* = 29^a^).

	Baseline	Post-intervention	*P*-value	Effect size (*d*)
CoP area (cm^2^)				
Single task	2.50 ± 2.43	2.08 ± 1.33	0.351	0.176
Dual task	3.83 ± 3.26	3.47 ± 3.43	0.430	0.149
CoP velocity (cm/s)				
Single task	1.29 ± 0.42	1.28 ± 0.50	0.889	0.026
Dual task	1.70 ± 0.69	1.90 ± 1.06	0.218	0.234

^a^ Due to corrupted/erroneous data for three participants, *n* = 29.

**Table 3:** Performance during the dual-tasks consisting of respectively 30 s of quiet standing and 10-m gait with a dual mathematical load (counting backwards of minus 7) at baseline and after 12 weeks of online dance training.

	Baseline	Post-intervention	*P*-value	Effect size (*d*)
Mathematical performance, quiet standing (*n* = 29^a^)				
Total # counted (*n*)	8.63 ± 3.76	9.05 ± 3.10	0.28	0.20
Correctly # counted (*n*)	7.63 ± 3.40	8.05 ± 3.00	0.307	0.22
Correctly # counted (%)	88 ± 10	89 ± 11	0.796	0.05
Mathematical performance, 10-m gait (*n* = 30^b^)				
Total # counted (*n*)	2.46 ± 0.92	2.72 ± 0.94	0.062	0.36
Correctly # counted (*n*)	2.14 ± 0.87	2.36 ± 0.99	0.146	0.27
Correctly # counted (%)	85 ± 20	85 ± 18	0.978	0.01

^a^ Due to corrupted/erroneous data for three participants, *n* = 29.

^b^ Due to knee injury at post-test (*n* = 1) and missing registration of counts (*n* = 1), *n* = 30.

### 10-m gait speed

**Figure 2:**
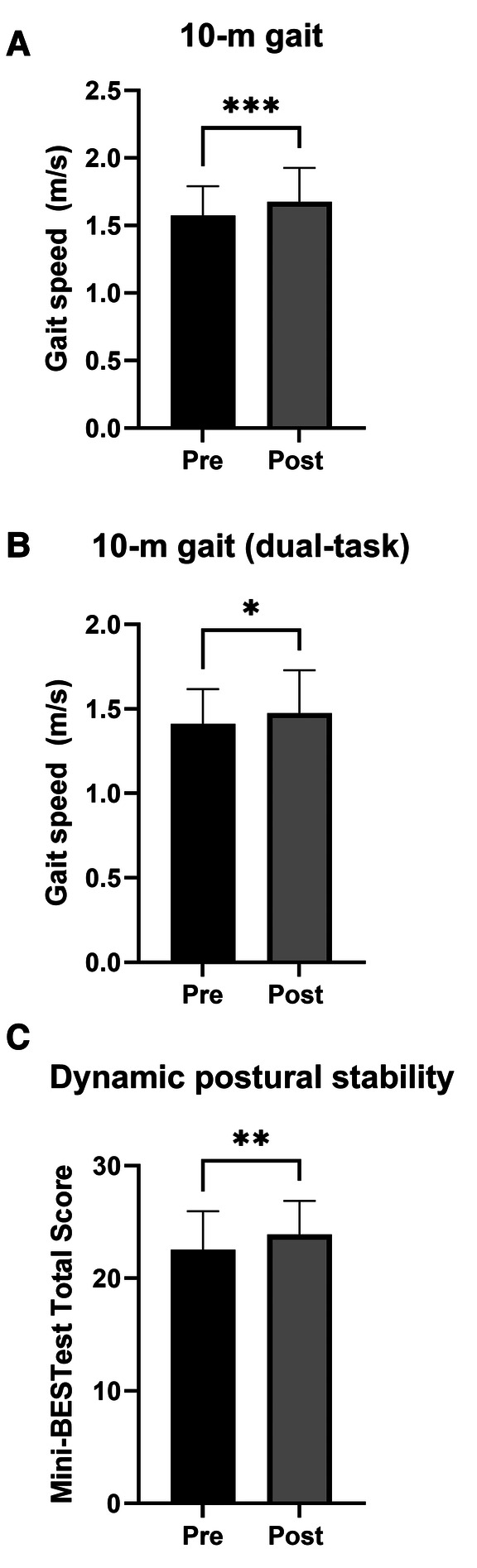
Mean gait speed and dynamic postural stability at baseline (Pre, black bars) and following 12 weeks of dance training (Post, grey bars). (**A**) 10-m gait speed. (**B**) 10-m gait speed with a mathematical dual-task. (**C**) Mini-BESTest total score. Mini-BESTest total score was calculated as the sum of the 14 test items. Note that for gait speed, *n* = 31. Error bars represent SD. Significant difference between baseline and post-intervention at *P < 0.05, **P < 0.01, and ***P < 0.001.

One participant was unable to complete the walking test at post-intervention, so for 10-m gait speed analyses is based on *n* = 31. 10-m gait speed was significantly faster after the 12 weeks of dance training (1.68 ± 0.25 m/s) compared to baseline (1.57 ± 0.22 m/s, *P* < 0.001, *d* = 0.737, Figure 2A). Also for 10-m gait speed with dual-task there was a statistically significant change from baseline (1.41 ± 0.21 m/s) to post-intervention (1.48 ± 0.25 m/s, *P* = 0.045, Figure 2B), yet with only a small effect size (*d* = 0.376). The mathematical performance during the dual-task is presented in Table 3.

### Dynamic postural stability

Dynamic postural stability evaluated by the Mini-BESTest total score was significantly improved at post-intervention (23.88 ± 3.01) compared to baseline (22.56 ± 1.41, *P* = 0.007, d = 0.52, Figure 2C).

## Discussion

When comparing the results after 12 weeks of the MT program with baseline values, our initial hypotheses were only partially confirmed: (I) static postural stability did not improve after the online dance intervention, as both the CoP area and velocity variables were not significantly different between before and after the intervention, (II) gait speed was faster post-intervention, both with and without a mathematical dual-task, although the magnitude of changes were small especially during the dual-task, and (III) dynamic postural control, determined via the Mini-BESTest, was enhanced following the intervention.

### Static postural stability

Assuming that static postural stability during a quiet standing task is achieved via controlling the body posture as if it behaves as an inverted pendulum swinging around the ankle joints (43), the optimal neuromuscular strategy to mitigate the effect of possible external perturbation is to increase the stiffness around the ankles by co-contracting the antagonistic and agonistic muscles around this joint (44). This strategy usually results in smaller and quicker displacements of the body after a perturbation, which, in laboratory settings, are usually represented by smaller areas and increased velocities of the CoP (45). Adding a supra-postural task, older adults would exhibit a decreased (I) static postural stability (46), or (II) performance of the supra-postural task when the postural task is highly demanding (13). Regardless of the addition or not of a supra-postural task, the present results did not show any significant change in CoP area or velocity nor in the performance of the dual-task intervention before/after. This could indicate: (I) a plateau effect, where the older women that participated in this study already presented good static postural stability prior to the intervention, (II) a sensitivity problem, where the force platform measurement used here where not able to detect the possible variations due to training, or (III) ineffective intervention for static postural stability since the dance training focused primarily on dynamic movements which relate to dynamic postural stability. While the level of static postural stability at baseline potentially could play a role, it seems unlikely that the force platform measurements did not provide the necessary sensitivity to detect changes in static postural stability, as the force platform used in the current study has a high sensitivity of ± 0.05%. What seems more likely to explain the lack of change, however, is a lack of specificity of the dance training, i.e., static balance may not have been sufficiently trained due to the dynamic nature of the salsa and contemporary dance styles.

### Dynamic postural stability

The ability to control the body position in space so its center of mass is within the base of support when the body suffers perturbation (dynamic balance) relies upon the available sensory information (usually vision, proprioceptive, and vestibular) to generate accurate motor responses to counter-react such perturbation and realign the vertical projection of the bodies' center of mass within the new base of support. Such a scenario is often encountered when performing daily life activities such as walking, running, dancing, carrying objects, using transportation, doing house chores, etc. The level of performance of such activities is usually associated with accidental falls in older adults, where lower independence in performing these activities is associated with a higher risk of fall accidents in older adults (47). After 12 weeks of the online dance training intervention, the Mini-BESTest score was significantly higher compared to baseline values, with a moderate effect size. These results indicate that the MT intervention was beneficial for improving the older women's dynamic postural stability. It is noteworthy that the baseline values for Mini-BESTest (average of 22.5) were below the cutoff value of 23 points for identification of fall risk for the average age (74 years) reported in the literature (48), which indicates that on average, participants were in a higher risk for fall accidents when enrolled in the study compared to their peers. After the intervention, the average score for this test increased to approximately 24 (with an average increase of 1.5 points), which is above the cutoff value for fall risk in that age group, and therefore suggests a meaningful change in dynamic postural control. However, it is important to highlight that the number of fall accidents where not monitored in this study, so the real effect on fall accidents from this intervention is yet to be investigated.

### Gait speed

Increasing gait speed after training has previously been correlated with lower fall risks in older adults. On average, the gait speed during the single-task increased 0.1 m/s after the present intervention, which even though representing a moderate change (d = 0.737) might not be clinically relevant. The lack of relevance of this result might be related to a ceiling effect. The maximum gait speed for older women is expected to be 1.7 m/s (49), while the average gait speed during the baseline single-task test was 1.5 m/s (all women). It is noteworthy that according to the latest recommendations (9), gait speed should be used for fall risk stratification among older adults. Indeed, gait speeds below 1 m/s are related to a higher risk of accidental falls in older adults (10), indicating that for our participants, gait speed would not be an efficient measure for fall risk stratification. Nevertheless, after the online dance intervention, our participants reached values close to the maximum (1.6 m/s), indicating that the intervention may have provided enough stimuli for motor improvement even though the participants already exhibited high performance values at baseline. Further studies should investigate whether an online training approach is also effective for less capable participants. Finally, although the statistically faster gait speed during the mathematical dual-task at post-intervention, compared to baseline, may point toward an enhancement of the participants' motor function under complex conditions, the clinical significance of this result is questionable as the magnitude of change was small (0.07 m/s, *d* = 0.376).

### Limitations

Despite the high degree of external validity in this study as the intervention was implemented in practice, in a municipal setting, the study is limited, however, by not including a control group for comparison. This limits the ability to discriminate between a real improvement in test performance due to the intervention, and increased familiarity with the tests due to the repeated testing, and thus the results from the current study should be interpreted with some caution. Also, no restrictions were made regarding prior dance experience or participation in other dance or training activities during the intervention period. Whilst this may have supported the inclusivity of study participants as well as reinforced the ecological validity of the study, it may also have affected the results in a way that complicates the ability to distinguish the benefits of the intervention from other activities. Moreover, the primary power calculation was based on a mental health outcome (i.e., self-reported loneliness), potentially limiting the robustness of conclusions made using traditional inferential statistics (paired *t*-tests) on these secondary outcomes. Another consideration is that our results might not be generalizable to older men, as only one man was included in the study, and he dropped out due to not feeling comfortable with being outnumbered. Such overwhelming female bias is consistent with the existing literature (23, 50) and should be addressed in future studies. Finally, this study is limited by the lack of control of participant attendance, and thus the data is not adjusted for adherence or the level of participation. As such, we also don't know whether cultural connections to the dance forms or accessibility concerns affected attendance, nor whether varying attendance rates and/or enjoyment affected the results. Due to our focus on inclusivity and implementation, participants were allowed to play and replay the recorded dance classes from YouTube on their own, and hence we were unable to accurately record attendance. This may have resulted in some participants attending every intervention session, while others may have not. While we acknowledge this limitation, it may have more ecological validity because it more closely approximates the real-life context of our study.

## Conclusion

In summary, while the MT online dance training intervention had a limited impact on static postural stability and gait speed, our data indicate important benefits of the intervention for dynamic postural control among older women. Further research, including studies with stricter control of study variables, is needed to investigate the efficacy of online dance training on the number of fall accidents and to explore its effectiveness with different participant groups.

## Data Availability

The raw data supporting the conclusions of this article will be made available by the authors, without undue reservation.

## Footnotes

^1^ https://www.youtube.com/@movingtogether8461
